# Fast Enhanced Exemplar-Based Clustering for Incomplete EEG Signals

**DOI:** 10.1155/2020/4147807

**Published:** 2020-05-08

**Authors:** Anqi Bi, Wenhao Ying, Lu Zhao

**Affiliations:** School of Computer Science and Engineering, Changshu Institute of Technology, Changshu, Jiangsu, China

## Abstract

The diagnosis and treatment of epilepsy is a significant direction for both machine learning and brain science. This paper newly proposes a fast enhanced exemplar-based clustering (FEEC) method for incomplete EEG signal. The algorithm first compresses the potential exemplar list and reduces the pairwise similarity matrix. By processing the most complete data in the first stage, FEEC then extends the few incomplete data into the exemplar list. A new compressed similarity matrix will be constructed and the scale of this matrix is greatly reduced. Finally, FEEC optimizes the new target function by the enhanced *α*-expansion move method. On the other hand, due to the pairwise relationship, FEEC also improves the generalization of this algorithm. In contrast to other exemplar-based models, the performance of the proposed clustering algorithm is comprehensively verified by the experiments on two datasets.

## 1. Introduction

Epilepsy is a common disease of nervous system, which is characterized by sudden brain dysfunction. Although there are many other neuroimaging modalities for the recognition of brain activity, EEG signals have a high temporal resolution which is up to the millisecond level, and its acquisition equipment is inexpensive, portable, and noninvasive. Nowadays, most diagnoses of epilepsy are based on clinical experience and the analysis of electroencephalogram (EEG) signals. Compared with manual diagnostic method, machine learning methods are less time-consuming and more consistent [1–6]. Specifically, many machine learning methods such as support vector learning [7, 8], Takagi–Sugeno–Kang (TSK) fuzzy system [9, 10], and Naïve Bayes [11] have been applied.

As we know that brain activity is a nonlinear, unstable complex network system, EEG signals we usually get are complicated. That is to say, some EEG signals are complete while others may miss some features, namely, incomplete. Therefore, recognition of epilepsy based on machine learning models will be more promising compared with clinical diagnosis depending on experience. Moreover, EEG signals have the characteristics of high dimension and stochasticity which limit the performance of most existing clustering models, such as k-means [11] and fuzzy *c* mean (fcm) [12]. K-means and fcm clustering models need to preset the number of clusters in advance. More specifically, the performance of the k-means model relies on the initialization of data, while the fcm model requires high interpretability. Thus, we focus on the exemplar-based clustering model [13] which is proposed by Frey in this paper. The exemplar-based clustering model has the advantages of automatically obtaining the cluster number, high efficiency, and not relying on the initialization of data.

**Figure 1:**
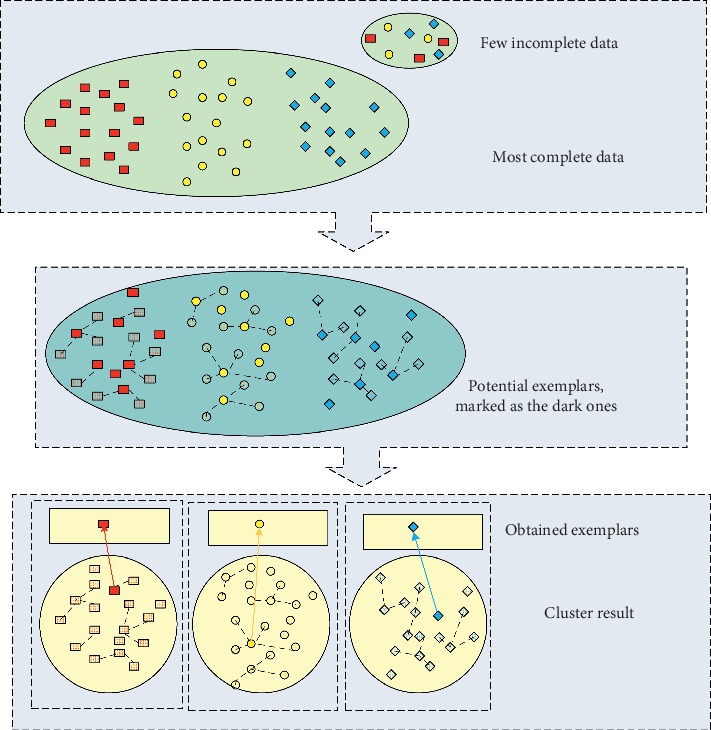
Clustering procedure of FEEC algorithm.

In conclusion, we consider the scenario of EEG signals consisting of most complete data and few incomplete data in this paper, as shown in Figure 1. Based on the previous work about the recognition of epileptic signals, we propose a novel fast enhanced exemplar-based clustering (FEEC) model for incomplete EEG signals. As shown in Figure 1, different from existing exemplar-based clustering models, FEEC compresses the exemplar list and reduces the pairwise similarity matrix, and then FEEC optimizes the target model by the enhanced *α*-expansion move framework. Moreover, the contributions of this paper can be highlighted as follows:

1. We extend the existing exemplar-based clustering algorithm into a fast version by compressing the potential exemplar lists in this study. FEEC compresses the number of potential exemplars by processing the most complete data in the first stage and extends the few incomplete data into the exemplar list. So, the complexity of FEEC is reduced as well.
2. Along with most existing exemplar-based clustering models, FEEC is built on the pairwise similarity matrix of data. Thus, after compression, FEEC would construct a new reduced similarity matrix, and the generalization of this algorithm is improved.
3. Moreover, this paper also considers the fact that the graph cuts [14] based optimization performs better than those loopy belief propagation (LBP) [15] based structure. So, the proposed FEEC algorithm optimizes the target model by the enhanced *α*-expansion move framework [16, 17].
4. Experimental results of both synthetic and real-world datasets indicate the promising efficiency of the proposed FEEC algorithm.

The rest of this paper is listed as follows. In Section 2, we introduce some static exemplar-based clustering models.  Section 3 discusses the proposed FEEC algorithm step by step. In Section 4, we analyze the experimental results and the comparison of FEEC and other existing methods.  Section 5 concludes this whole paper.

## 2. Background

Since EEG signal feature extraction methods and exemplar-based clustering models are two important supporting theories for the FEEC model in this study, we will briefly introduce several feature extraction methods and exemplar-based clustering models in this section.

### 2.1. Feature Extraction Methods

Original EEG signals have the characteristics of high dimensionality, stochasticity, and nonlinearity. It would be computationally very expensive to extract features from raw EEG signals; nowadays, many feature extraction methods have been proposed to handle this problem. In sum, there are three categories, i.e., time-domain features, frequency-domain features, and time-frequency features.

More specifically, in time-domain analysis, statistics component features of the raw EEG signals will be analyzed [18]. In frequency-domain analysis, power spectrum analysis and short-time Fourier transform (STFT) [19, 20] are commonly used. In time-frequency analysis, time and frequency domain are simultaneously extracted from nonstationary EEG signals. Wavelet and other improved versions [21, 22] are widely used in EEG signal processing. We utilize KPCA to extract feature in this paper.

### 2.2. Exemplar-Based Clustering Models

Exemplar-based clustering models select cluster centers, namely, exemplars, from existing actual data. We focus on exemplar-based clustering models in this paper and briefly introduce affinity propagation (AP) [13] and enhanced *α*-expansion move (EEM) [17] in this section. And several extended versions for different scenarios are shown in Table 1. The target fucntion defined by exemplar-based clustering model equals to the minimization problem of energy function of Markov random field(MRF). Two existing optimization startegies have been utilized and evolved into AP and EEM frameworks accordingly. Moreover, loopy belief propagation (LBP) [23] is used in AP, while graph cuts technique [15] is used in EEM, respectively.

#### 2.2.1. Affinity Propagation

AP is based on message passing among data points, and its target function is defined as follows:

(1) $$\begin{array}{c} \underset{E}{\max}\sum_{p=1}^{N}S\left(i,k\right)-\sum_{p=1}^{N}\delta_{p}\left(E\right), \end{array}$$

where

(2) $$\begin{array}{c} \delta_{p}\left(E\right)=\left\{\begin{array}{cc} \infty , & \text{if }E\left(x_{p}\right)\neq p,\text{but }\exists x_{q}:E\left(x_{p}\right)=p,\\ 0, & \text{otherwise,} \end{array}\right. \end{array}$$

where **X**={*x*_1_, *x*_2_,…, *x*_*N*_} ∈ *ℝ*^*N∗D*^ is an input dataset and *N* is the total number of *D*-dimensional data points. **S***E* is the output of this framework, and the element *E*(*x*_*p*_) is referred to the exemplar for each *x*_*p*_.

According to AP, each point receives availability message **A**(*i*, *k*) and sends responsibility **R**(*i*, *k*) message simultaneously, which are defined as follows:

(3) $$\begin{array}{c} R\left(i,k\right)=S\left(i,k\right)-\underset{j,\text{s}.\text{t}.\;j\neq k}{\text{max}}\left\{A\left(i,j\right)+S\left(i,j\right)\right\}, \end{array}$$

(4) $$\begin{array}{c} A\left(i,k\right)=\left\{\begin{array}{cc} \text{min}\left\{0,R\left(k,k\right)\right.+\underset{j,\text{s}.\text{t}.j\neq \left\{i,k\right\}}{\sum }\left\{\text{max}\left(0,R\left(j,k\right)\right)\right\}, & i\neq k,\\ \underset{j,\text{s}.\text{t}.\;j\neq \left\{i,k\right\}}{\sum }\left\{\text{max}\left(0,R\left(j,k\right)\right)\right\}, & i=k, \end{array}\right. \end{array}$$

where **S** is the similarity matrix of data points and is defined as **S**(*i*, *j*)=−‖**x**_*i*_ − **x**_*j*_‖^2^. Meanwhile, **S**(*k*, *k*)=*p* where *p* is named as preferences in this framework. Moreover, its value should be independent and can be set to a constant.

AP does not require presetting the number of the cluster and the performance is stable. Considering these advantages, many extended versions of AP have been proposed [24, 25]. Specifically, AP defines fading factor to adjust the iteration speed; adAP [24] is proposed to determine this fading factor adaptively. Moreover, several extended versions of AP methods have been proposed to deal with large data and link constraints. For instance, IAPKM, IAPNA, and IAPC [26, 27] employ incremental strategy and semisupervised AP and SSAP [28] concentrate on instance-level constraints. A two-stage fast version of AP (FAP) [29] is also proposed to improve the efficiency. However, although AP has been obtaining its success in various applications, when we attempt to directly apply AP to incomplete EEG signals, the performance is unsatisfactory.

#### 2.2.2. Enhanced *α*-Expansion Move

In 2014, Zheng and Chen [17] utilized enhanced *α*-expansion move framework to optimize the object function of exemplar-based clustering models and accordingly proposed the EEM clustering model. In line with the mathematical symbols above, the target function of EEM is defined as follows:

(5) $$\begin{array}{c} \underset{E}{\text{max}}\sum_{p=1}^{N}s\left(x_{p},x_{E\left(x_{p}\right)}\right)-\sum_{p=1}^{N}\sum_{q>p}^{N}\theta_{p,q}\left(E\left(x_{p}\right),E\left(x_{q}\right)\right), \end{array}$$

where

(6) $$\begin{array}{c} \theta_{p,q}\left(E\left(x_{p}\right),E\left(x_{q}\right)\right)=\left\{\begin{array}{cc} M,E\left(x_{p}\right)=q,E\left(x_{q}\right)\neq q,\text{ or }E\left(x_{q}\right)=p, & E\left(x_{p}\right)\neq p,\\ 0, & \text{otherwise,} \end{array}\right. \end{array}$$

(7) $$\begin{array}{c} \theta_{p,q}\left(E\left(x_{p}\right),E\left(x_{q}\right)\right)+\theta_{p,q}\left(\alpha ,\alpha \right)\leq \theta_{p,q}\left(E\left(x_{p}\right),\alpha \right)+\theta_{p,q}\left(\alpha ,E\left(x_{q}\right)\right). \end{array}$$

In terms of [17], *α*-expansion move algorithm has been proved to be effective in the optimization of the target function equation (5). Specifically, when ∀*p*, *q*, *E*(**x**_*p*_), *E*(**x**_*q*_),  *α* ∈ {1,2,…*N*}, equation (7) is verified. Furthermore, according to graph theory, in the fast *α*-expansion move algorithm, the expansion range is limited in a one exemplar. To break this limit, the EEM model enlarges the range to the whole exemplar set *E* when optimizing and defines a second exemplar *S*(*i*) for each point **x**_*i*_ as follows:

(8) $$\begin{array}{c} S\left(i\right)=\underset{s\in \left(E/l\right)}{\text{argmax}}d\left(x_{i},x_{s}\right),\;\forall x_{i}\in X_{l}, \end{array}$$

where **X**_*l*_={**x**_*i*_|*E*(**x**_*i*_=*l*)} is the dataset among which the exemplar is *l* and *s* ∈ (*E*/*l*) represents other exemplars in *E* except for *l*.

The EEM clustering model is a state-of-the-art achievement of exemplar-based clustering model and has been proved to be efficient and effective for numerous scenarios [16, 17, 30]. IEEM [30] is proposed to deal with link constraints by embedding a bound term in the target function. For dynamic data stream, Bi and Wang [16] also proposed an incremental EEM version DSC which processes data chunk by chunk. However, for incomplete EEG signals, these methods would not recognize epilepsy well.

## 3. Fast Enhanced Exemplar-Based Clustering Model

In this section, the proposed FEEC model will be stated and theoretically analyzed in detail. We first compress the exemplar list and reduce the pairwise similarity matrix, and then the target model is optimized by the enhanced *α*-expansion move framework.

### 3.1. Framework

**Figure 2:**
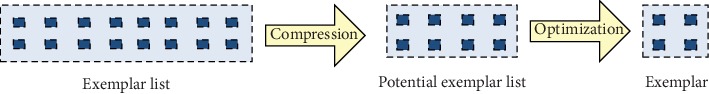
Framework of FEEC algorithm: compression stage and optimization stage.

As mentioned in the introduction section, we focus on the incomplete EEG signals which consist of most complete data and few incomplete data. To improve the efficiency of the EEM clustering model for these incomplete EEG signals, the proposed FEEC framework includes two stages, namely, compression stage and optimization stage. As shown in Figure 2, the compression stage compresses the potential exemplar list and the optimization stage determines the optimal exemplars from the potential exemplar list. Accordingly, the target function can be defined as follows:

(9) $$\begin{array}{c} \underset{E}{\max}\sum_{i=1}^{N}s\left(x_{i},x_{E\left(x_{i}\right)}\right)-\sum_{i=1}^{N}\eta_{i}\left(E\right), \end{array}$$

where **X** = [**X**_*c*_, **X**_*l*_] is the input dataset consisting of most complete data **X**_*c*_ = {**x**_*c*,1_, **x**_*c*,2_,…, **x**_*c*,*N*_*c*__} and few incomplete data **X**_*l*_ = {**x**_*l*,1_, **x**_*l*,2_,…, **x**_*l*,*N*_*l*__}. The total number of data is defined as *N* = *N*_*c*_ + *N*_*l*_, where *N*_*c*_ and *N*_*l*_ are the number of complete and incomplete data, respectively. Remember that we only consider the scenario that *N*_*c*_ ≫ *N*_*l*_ in this study. The second term in equation (9) guarantees the validity of the exemplar list; its definition is similar to that of **δ** in equation (2). In the end, *E* = {*E*(**x**_1_), *E*(**x**_2_),…, *E*(**x**_*N*_)} represents the exemplar set in question.

In the compression stage, the number of potential exemplar list will be reduced by exemplar-based selection algorithm, namely, EEM method in this study. To be specific, we apply the EEM model on the most complete data to obtain the potential exemplars for these data. FEEC also pulls the few incomplete data into this potential exemplar list and then constructs compressed similarity matrix. Therefore, after compression, only the pairwise similarities between data and potential exemplars would be preserved. Considering that the FEEC method is built on the pairwise similarity matrix, the following clustering procedure would be applied on this compressed similarity matrix. Furthermore, the scale of similarity matrix is reduced from *N*^2^ to *Nc*, where *N* and *c* are the number of data and potential exemplars, respectively.

In the optimization stage, only the similarity relationship between data and potential exemplars is considered. The new target function after compression is similar to that of other exemplar-based clustering model, like equations (1) and (5), so we take graph cuts and LBP into account. Nevertheless, graph cuts based optimization framework outperforms LBP structure [31]. So, the proposed FEEC utilizes the *α*-expansion move method to optimize the new target function. Moreover, along with EEM, FEEC also expands the expansion move space from a single data to the second optimal exemplar.

### 3.2. Compression Stage

In the compression stage, the target function of complete data can be defined as follows:

(10) $$\begin{array}{c} \underset{E_{c}}{\min}\sum_{i=1}^{N_{c}}d\left(x_{c,i},x_{E_{c}\left(x_{c,i}\right)}\right)+\sum_{i=1}^{N_{c}}\sum_{j>i}^{N_{c}}\theta_{i,j}\left(E_{c}\left(x_{c,i}\right),E_{c}\left(x_{c,j}\right)\right), \end{array}$$

where **X**_*c*_={**x**_*c*,1_, **x**_*c*,2_,…, **x**_*c*,*N*_*c*__} ∈ *ℝ*^*N*_*c*_*∗D*^ is the complete *D*-dimensional data and *N*_*c*_ is the number of these data. *E*_*c*_ is the potential exemplar list for most complete data, and the element among *E*(**x**_*c*,*i*_) is referred to the potential exemplar for each **x**_*c*,*i*_. The optimization framework of other exemplar-based clustering models, like EEM, can be utilized to solve equation (10). In this paper, we select the graph cuts algorithm instead of message-passing algorithm to compress the potential exemplar list. Thus, the potential exemplar list for complete data *E*_*c*_ can be determined, and the number of potential exemplars is defined as *c*_*c*_.

The potential exemplar list after compression stage would be

(11) $$\begin{array}{c} E_{\text{new}}=\left[E_{c},E_{l}\right], \end{array}$$

where *E*_*l*_ is the exemplar set for the few incomplete data, which is the incomplete data itself actually. That is to say, *E*_*l*_(**x**_*l*,*i*_)=*i*.

In this stage, we reduced the number of potential exemplars from *N*_*c*_ to *c*_*c*_. In terms of the analysis in [13, 17, 30], the time complexity of this stage will be *O*(*N*_*c*_^2^). Compared with the time complexity *O*(*N*^2^), if we apply exemplar-based clustering model directly considering the fact that *N* < *N*_*c*_, the time complexity of this compression algorithm would be acceptable.

Therefore, on the basis of the new exemplar list after compression, we can construct the new similarity matrix **S**_new_ ∈ *ℝ*^*N*×*c*^; the element relates to the distance, namely, **S**_new_(*i*, *j*)=−‖**x**_*i*_ − **x**_*E*_new_(*j*)_‖^2^. The scale of the similarity matrix reduces from *N*^2^ to *Nc*, where *c*=*c*_*c*_+*N*_*l*_ represents the number of potential exemplars.

### 3.3. Optimization Stage

After compression, we define the new target function as follows:

(12) $$\begin{array}{c} \underset{E}{\max}\sum_{i=1}^{N}\sum_{j=1}^{c}S_{\text{new}}\left(i,j\right)-\sum_{i=1}^{N}\eta_{i}\left(E\right), \end{array}$$

where **S**_new_ is the new similarity matrix constructed after compression.

In this section, we construct an optimization framework for equation (12). The second term of equation (12) is set to guarantee the validity of the exemplar list; in order to utilize the graph cuts based method, this term should be pairwise [17]. So, *η*_*i*_(*E*) is modified as *η*_*i*,*j*_(*E*). Furthermore, similar to equation (5), we define *η*_*i*,*j*_(*E*) as follows:

(13) $$\begin{array}{c} \eta_{i,j}\left(E\right)=\left\{\begin{array}{cc} M,E\left(x_{i}\right)=j,E\left(x_{j}\right)\neq j,\text{ or }E\left(x_{j}\right)=i, & E\left(x_{i}\right)\neq i,\\ 0, & \text{otherwise}. \end{array}\right. \end{array}$$

It has been proved that with the definition of *η*_*i*,*j*_(*E*), equation (12) can be optimized by the enhanced *α*-expansion method [30]. To improve the efficiency of framework, this method enlarges the expansion move to the second optimal exemplar.

Before optimization, we explain several symbols involved. First, we define **X**_*e*_ as those data with exemplar **x**_*e*_ and **x**_*α*_ as the current potential exemplar. Then, the enhanced *α*-expansion move method considers the second optimal exemplar, which is defined as

(14) $$\begin{array}{c} S\left(x_{i}\right)=\underset{s\in \left(E/\alpha \right)}{\text{argmax}}S_{\text{new}}\left(x_{i},x_{s}\right),\;\forall x_{i}\in X_{\alpha }, \end{array}$$

where (*E*/*α*) is the potential exemplar list except for *α*.

**Figure 3:**
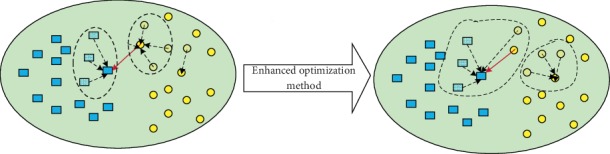
Case (i): **x**_*α*_ is an exemplar.

**Figure 4:**
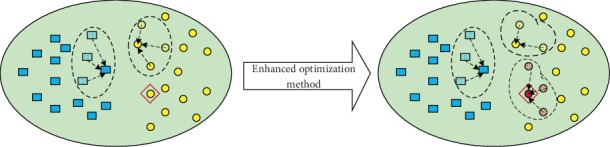
Case (ii): **x**_*α*_ is not an exemplar.

Apparently, this optimization method should consider two cases, namely, **x**_*e*_ is among exemplar list or not, as shown in Figures 3 and 4. To be specific, Figure 3 illustrates the case when **x**_*α*_ is an exemplar, while Figure 4 shows the case when **x**_*α*_ is not an exemplar. Remember that only when **x**_*α*_ is a potential exemplar, *S*(**x**_*i*_) works. We utilize the concepts of “energy reduction” because this method was first used to optimize the Markov random field (MRF) energy function.

In the situation shown in Figure 3, either ∀**x**_*i*_ ∈ **X**_*α*_ changes its exemplar to *S*(**x**_*i*_) or nothing is changed. Therefore, the energy reduction *R*1 would be defined as

(15) $$\begin{array}{c} R1=\text{max}\left(0,R1_{\alpha }\right), \end{array}$$

where *R*1_*α*_ is the energy reduction when ∀**x**_*i*_ ∈ **X**_*α*_ changes its exemplar to *S*(**x**_*i*_) and is defined as

(16) $$\begin{array}{c} R1_{\alpha }=\sum_{x_{i}\in X_{\alpha }}\left(S_{\text{new}}\left(x_{i},S\left(x_{i}\right)\right)-S_{\text{new}}\left(x_{i},x_{\alpha }\right)\right). \end{array}$$

On the other hand, as shown in Figure 4, a new exemplar **x**_*α*_ should be considered. Whether to accept the new exemplar is decided by the energy reduction *R*2, which will be discussed next. First, we assume a new exemplar **x**_*α*_ is accepted. In fact, the following procedure is similar to that shown in Figure 3. Specially, the remaining data would change its exemplar to either **x**_*α*_ or *S*(**x**_*i*_). For data in cluster *e* ∈ *E*, theoretical analysis proves that only when the exemplar **x**_*e*_ changes its exemplar, ∀**x**_*i*_ ∈ **X**_*e*_ would change its exemplar as *S*(**x**_*i*_). In this case, the energy reduction is defined as follows:

(17) $$\begin{array}{c} R2_{e}=\sum_{x_{i}\in X_{e}}\left(S_{\text{new}}\left(x_{i},S\left(x_{i}\right)\right)-S_{\text{new}}\left(x_{i},x_{e}\right)\right). \end{array}$$

Otherwise, some data in cluster *e* may change their exemplar as **x**_*α*_; we define these data as **X**_*e*,*α*_^/*e*^, and the corresponding energy reduction *R*2_*e*_ is defined in the following equation:

(18) $$\begin{array}{c} R2_{\alpha }=\sum_{x_{i}\in X_{e,\alpha }^{/e}}\left(S_{\text{new}}\left(x_{i},x_{\alpha }\right)-S_{\text{new}}\left(x_{i},x_{e}\right)\right). \end{array}$$

Then, the energy reduction *R*2 is defined as follows:

(19) $$\begin{array}{c} R2=S_{\text{new}}\left(x_{\alpha },x_{\alpha }\right)-S_{\text{new}}\left(x_{\alpha },x_{E_{\text{new}}\left(\alpha \right)}\right)+\text{max}\sum_{e\in E}\left\{R2_{e},R2_{\alpha }\right\}. \end{array}$$

In sum, the new target function equation (12) is optimized, and the optimal exemplar list for the EEG signals is generated.

### 3.4. Time Complexity and Description

The similarity relationship can be measured by Euclidean distance between data, defined as *d*(**x**_*i*_, **x**_*j*_) in this study. The proposed algorithm FEEC consists of two stages, namely, compression stage and optimization stage. After compression, the scale of similarity matrix reduces from *N*^2^ to *Nc*, so the optimization stage has the time complexity of *O*(*c*^2^). Therefore, the complexity of FEEC is considerably promising.

Based on the theoretical analysis above, the proposed FEEC for incomplete data can be summarized as Algorithm 1.

## 4. Experimental Study

To comprehensively evaluate the proposed algorithm FEEC, we have conducted several experiments based on both synthetic and real datasets. We also compare our new model with basic exemplar-based clustering model, namely, AP and EEM; to show these experimental results, we choose four performance indices in this section. In our experiments, all the algorithms were implemented using 2010a Matlab on a PC with 64 bit Microsoft Windows 10, an Intel(R) Core(TM) i7-4712MQ, and 8 GB memory.

### 4.1. Data Preparation

**Figure 5:**
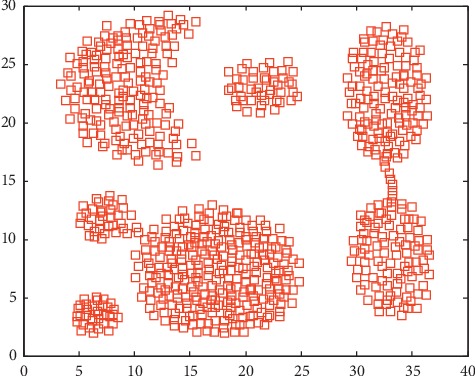
Description of Aggregation data.

**Figure 6:**
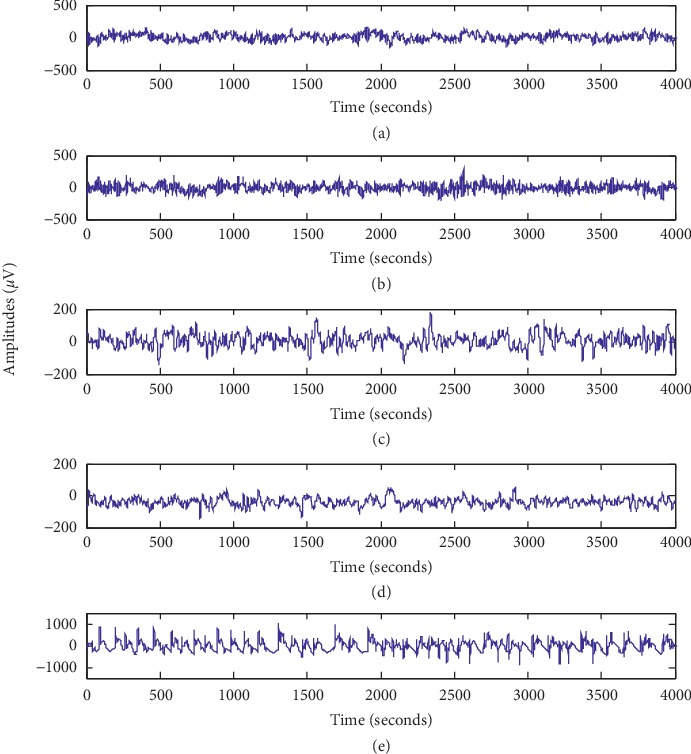
Typical EEG signals in groups A–E.

We choose Aggregation [32], as shown in Figure 5, and the Bonn EEG signal datasets in this section. The Bonn dataset [9, 10] is from the University of Bonn, Germany (http://epileptologie-bonn.de/cms/upload/workgroup/lehnertz/eegdata.html). The EEG dataset contains five groups (A to E and each group contains 100 single channel EEG segments of 23.6s duration. The sampling rate of all the datasets was 173.6 Hz.  Figure6 shows five healthy and epileptic EEG signals, and Table 2 lists detailed descriptions of these signals.  Table 3 shows a brief description of these datasets. To construct the incomplete data scenario, we randomly choose 80% data as complete data and the remaining 20% as the incomplete data. We utilize KPCA to extract features from EEG signals in this section.

### 4.2. Performance Indices

Here, we give the definitions of the three adopted performance indices ENERGY, NMI, and accuracy. Along with the description in [12, 16, 30, 33, 34], we call the result outputted by these involved models as cluster and the true labels as class.

#### 4.2.1. ENERGY

Since all the mentioned clustering algorithms are optimized, respectively, by the energy functions of the same type, we can compare them in terms of their energy values, defined as follows:

(20) $$\begin{array}{c} \text{ENERGY}=\sum_{k}\sum_{i}d\left(x_{k},x_{k,i}\right), \end{array}$$

where **x**_*k*_ denotes the *k*th exemplar, **x**_*k*,*i*_ is the *i*th data point in *k*th cluster, and *d*(**x**_*k*_, **x**_*k*,*i*_) is the Euclidean distance between **x**_*k*_ and **x**_*k*,*i*_ which can be seen as a measurement of energy.

#### 4.2.2. NMI

NMI has been widely used to evaluate the clustering quality as well, and its value can be calculated by the following equation:

(21) $$\begin{array}{c} \text{NMI}=\frac{\sum_{i=1}^{\left|C\right|}\sum_{j=1}^{\left|C\right|}N_{i,j}\text{ln}\left(NN_{i,j}/N_{i}N_{j}\right)}{\sqrt{\left(\sum_{i=1}^{\left|C\right|}N_{i}\text{ln}\left(N_{i}/N\right)\right)\left(\sum_{j=1}^{\left|C\right|}N_{j}\text{ln}\left(N_{j}/N\right)\right)}}, \end{array}$$

where *N*_*i*,*j*_ is how clusters fit the classes, *N*_*i*_ is the number of data points in *i*th cluster, *N*_*j*_ is the number of data in *j*th class, and *N* is the total number of data points.

#### 4.2.3. Accuracy

Accuracy is a more direct measure to reflect the effectiveness of clustering algorithms, which is defined as

(22) $$\begin{array}{c} \text{accuracy}=\frac{\sum_{i=1}^{N}\delta \left(c_{i},\text{map}\left({\hat{c}}_{i}\right)\right)}{N}, \end{array}$$

where *c*_*i*_ is the real label of data points and ${\hat{c}}_{i}$ is the obtained clustering label. *δ*(*i*, *j*)=1, if *i*=*j*; *δ*(*i*, *j*)=0, otherwise. Function map(·) maps each obtained cluster to real class, and the optimized mapping function can be found in Hungarian algorithm.

The values of NMI and Acc range from 0 to 1, and the more it is close to 1, the more effective the clustering algorithm is. What is worth to mention is that we put % in the following relevant tables to show better precision. As to the performance index ENERGY, the smaller the value is, the better the clustering algorithm is.

### 4.3. Experimental Results and Discussion

**Figure 7:**
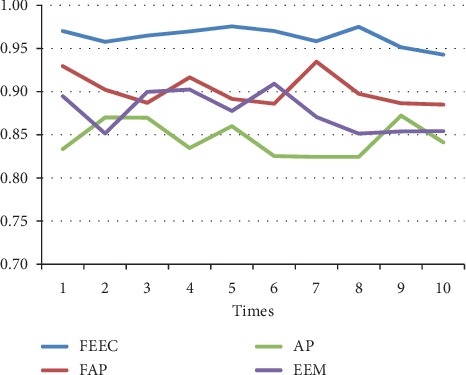
Comparison of NMI on Aggregation dataset.

**Figure 8:**
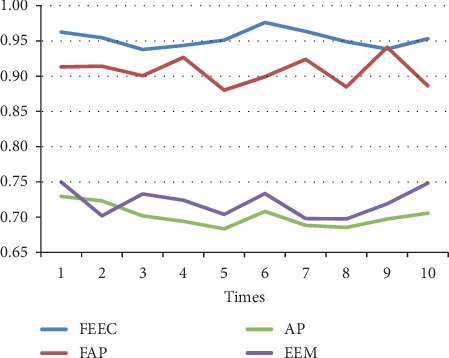
Comparison of accuracy on Aggregation dataset.

**Figure 9:**
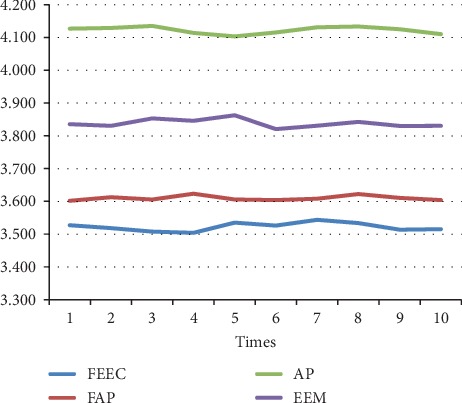
Comparison of accuracy on Aggregation.

**Figure 10:**
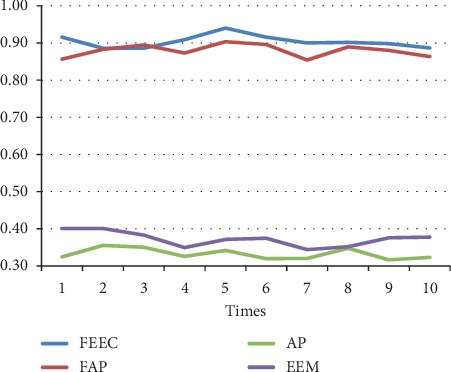
Comparison of NMI on Bonn epileptic EEG signals.

**Figure 11:**
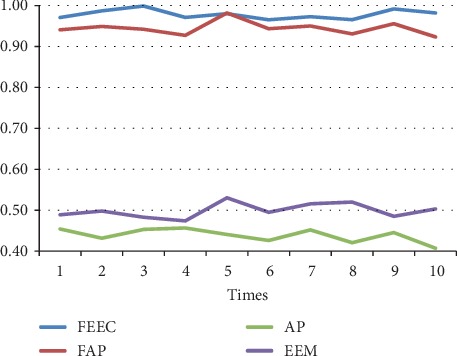
Comparison of accuracy on Bonn epileptic EEG signals.

**Figure 12:**
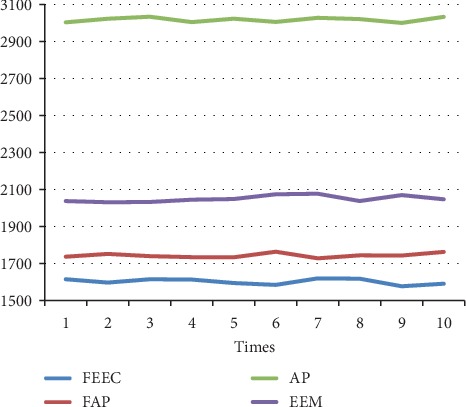
Comparison of ENERGY on Bonn epileptic EEG signals.

The parameters involved FAP, AP, and EEM are in line with [13, 17, 29]. The preference *s*(*i*, *i*) is set to be the median value of similarities between data. We run each algorithm over 10 runs under same parameters; the average results are shown in Table 4. Moreover, the detailed comparison in terms of the above 3 terms, NMI, accuracy, and ENERGY, are shown in Figures 7–12 and Table 4, respectively.

By analyzing Figures 7–11 and Table 4 in detail, we can conclude the following:

1. The proposed algorithm FEEC can cluster data with 80% complete data and 20% incomplete data, and in most cases, the performance is very convincing. Specifically, for both Aggregation and epileptic EEG signals, FEEC performs best, in terms of NMI, accuracy, and ENERGY.
2. As to the computational time, compared with EEM, FEEC takes less time. Thus, with the assistance of compression stage, the time complexity of FEEC has been reduced, and the efficiency is improved as well. And FEEC has an equivalent computational time with FAP. Comparing other criteria of FEEC and FAP, it is worthwhile to spend more time.
3. The proposed FEEC needs no more parameters except for preferences, while the performance of FAP relies much on *k*, which determines the number of nearest exemplars. Accordingly, in terms of the involved datasets in this section, FEEC would achieve satisfactory clustering results.

## 5. Conclusions

The diagnosis and treatment of epilepsy is always a significant direction for both machine learning and brain science. This paper newly proposes a fast exemplar-based clustering method for incomplete EEG signal. The FEEC method includes two stages, namely, compression and optimization. The performance of the proposed clustering algorithm is comprehensively verified by the experiments on two datasets.

Although most recognition methods of epilepsy are based on EEG signals at present, researchers also have to study on other neuroimaging modalities, such as cortical electroencephalography (ECoG), functional infrared optical imaging (fNIR), functional magnetic resonance imaging (fMRI), positron emission tomography (PET), and magnetoencephalography (MEG). Considering the fact that the brain activity is a nonlinear, networked, and unstable complex system, we would focus on the multimodal clustering model for these neuroimaging modality signals in future.

## Figures and Tables

**Algorithm 1:**
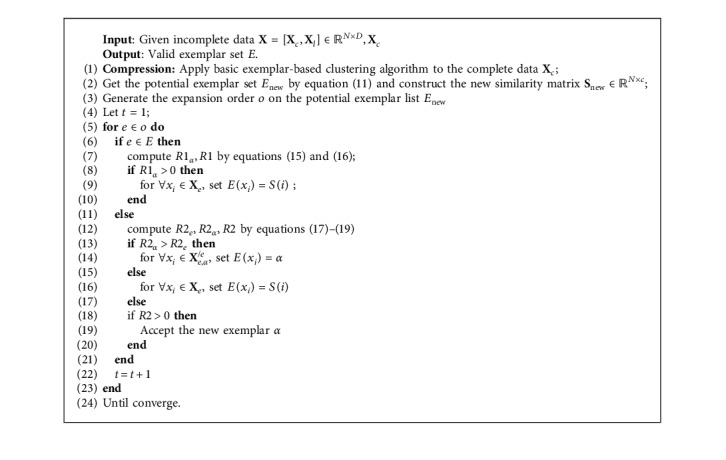
Fast enhanced exemplar-based clustering algorithm.

**Table 1:** Descriptions of several state-of-the-art exemplar-based algorithms.

	Optimization	Extended version	Descriptions
AP	Message-passing	adAP	Determine fading factor adaptively
		IAPKM, IAPNA, IAPC	Incremental version for data stream
		SAP,SSAP	Deal with instance-level constraints
		FAP	Two-stage fast version

EEM	Graph cuts	DSC	Dynamic data stream cluster
		IEEM	Deal with link constraints
		FEEC (newly proposed)	Two-stage fast version

**Table 2:** Description of healthy and epileptic EEG data.

Subjects	Groups	Size of data	Descriptions
Healthy	A	100	Signals captured from volunteers with eyes open
	B	100	Signals captured from volunteers with eyes closed

Epileptic	C	100	Signals captured from volunteers during seizure silence intervals
	D	100	Signals captured from volunteers during seizure silence intervals
	E	100	Signals captured from volunteers during seizure activity

**Table 3:** Brief description of Aggregation and Bonn Epileptic EEG signals.

Datasets	Number of objects	Number of attributes	Number of clusters
Aggregation	788	2	7
Bonn	500	6	5

**Table 4:** Average experimental results over 10 runs on Aggregation and Bonn epileptic EEG signals.

Dataset	Algorithms	NMI	Accuracy	ENERGY	Time
Aggregation	FEEC	0.9636	0.9530	3522.38	9.54
	FAP	0.9017	0.9130	3609.79	9.65
	AP	0.8455	0.7016	4122.64	28.59
	EEM	0.8765	0.7208	3838.29	16.804

Bonn epileptic EEG signals	FEEC	0.9038	0.9786	1602.87	3.75
	FAP	0.8794	0.9445	1744.22	3.65
	AP	0.3324	0.4387	3018.12	3.97
	EEM	0.3728	0.4991	2050.51	6.01

## Data Availability

The data used to support the findings of this study are available from the corresponding author upon request.
